# Design and Experimental Evaluation of a Dual-Cantilever Piezoelectric Film Sensor with a Broadband Response and High Sensitivity

**DOI:** 10.3390/mi14112108

**Published:** 2023-11-17

**Authors:** Wei Xin, Zhaoyang He, Chaocheng Zhao

**Affiliations:** College of Information Science and Technology, Beijing University of Chemical Technology, Beijing 100029, China; 2022210498@buct.edu.cn (Z.H.); 2020210504@buct.edu.cn (C.Z.)

**Keywords:** PVDF piezoelectric film, cantilever beam, dual-arm structure, natural frequency, measurement bandwidth, sensitivity

## Abstract

Cantilever-beam-type PVDF (Polyvinylidene Fluoride) piezoelectric film sensors are commonly utilized for vibration signal detection due to their simple structures and ease of processing. Traditional cantilevered PVDF piezoelectric film sensors are susceptible to the influence of the second-order vibration mode and have a low lateral stress distribution at the free end, which limit their measurement bandwidth and sensitivity. This study is on the design of a dual-cantilever PVDF piezoelectric film sensor based on the principle of cantilevered piezoelectric film sensors. The results of the experiments indicate that, compared to a typical single-arm piezoelectric cantilever beam vibration sensor, the developed sensor has a longer second-order natural frequency that ranges from 112 Hz to 453 Hz, while the first-order natural frequency is maintained at around 12 Hz. This leads to a better ratio of the second-order natural frequency to the first-order natural frequency and a wider frequency response range. At the same time, the sensitivity is increased by a factor of 3.48.

## 1. Introduction

As a physical phenomenon, vibration signals have multiple practical applications. Vibration signals are often measured and analyzed to extract information about systems, equipment, or structures. This information is utilized for applications such as fault detection [1], equipment performance optimization and preventative maintenance [2], human health monitoring [3], structural health monitoring [4,5], as well as vibration energy harvesting [6]. Due to the extremely weak, primarily low frequency and wide vibration frequency range of the generated vibration signals in earthquake exploration, water pipeline leakage, and bridge structure monitoring industries [7,8,9], vibration sensors with a high sensitivity and wide measurement bandwidth have increasingly significant application potential to ensure the accuracy and reliability of risk assessments and safety monitoring.

Piezoelectric cantilever beams are crucial sensitive components that convert vibrational signals into electrical signals. They possess notable characteristics such as a rapid frequency response, a high strength, and low natural frequencies [10,11,12]. PVDF (Polyvinylidene Fluoride) is a high-performance polymer with exceptional piezoelectric characteristics, such as a high piezoelectric coupling coefficient, good chemical stability, and a wide frequency response range [13,14,15]. Compared with piezoelectric ceramic materials, the advantages of PVDF are that it is highly flexible and less likely to fracture [16]. As a result, it can more easily adapt to cantilever beam construction, making it a typical sensitive measuring element in vibration sensors [17].

In recent years, many researchers have made improvements to the structure of piezoelectric cantilever beams in order to measure mechanical vibrations in specific environments. Li, Y. et al. [18] designed a circular-structured piezoelectric film cantilever beam to reduce the dimensions of the structure and increase the charge output. This design achieved a stable sensitivity of 230 mV/(m/s^2^) and reduced the lower measurement frequency limit to 4 Hz. Abdul, B. et al. [19] used piezoelectric cantilever beam construction to create an omni-directional response hydrophone. A first-order natural frequency of 20 kHz and a maximum sensitivity of 153 dB were obtained by varying the length of the cantilever beam between 100 and 1000 μm. Singh, R.K. et al. [20] created a PVDF nanofiber vibration sensor by taking advantage of its flexibility and low weight. Within the prescribed strain range, the linear voltage and strain relationship was determined and vibration measurements were performed on the pipe string. Bhuvana, S. et al. [21] found that the highest voltage produced by a diesel engine with a vibration frequency of 73 Hz is 2.45 mV when measured using the specified vibration sensor with a natural frequency of 80 Hz according to the design and analysis of the cantilever piezoelectric vibration sensor. Yi, J. et al. [22] mathematically modeled and experimentally studied the stress generated by PVDF film deformation. This research was successfully applied to deformation detection in car tires and measurements of insect motion. Chauhan, S. and Ansari, M.Z. [23] improved piezoelectric cantilever sensors’ performance by introducing holes in its profile at different locations. Daku, B.L. et al. [24] designed a low-frequency acceleration vibration sensor with a measurement frequency band of 10–100 Hz, which was successfully applied in mining vibration measurements. Rougeot, P. et al. [25] presented a piezoelectric actuator with an embedded piezoelectric sensor for micromanipulation and microassembly tasks, and the results showed that the PVDF sensing element can track the actuation performance.

However, due to the free-end mass block, traditional PVDF piezoelectric film cantilever beam structures exhibit bending deformations during vibration, causing the second-order frequency mode shape to often occur at approximately ten times the frequency of the first-order mode shape [26]. The measuring frequency bandwidth is limited owing to the superposition effect of the second-order vibration modes [27]. 

In this work, based on the principle of cantilever-beam-type piezoelectric thin film sensing, a dual-arm PVDF piezoelectric film cantilever beam structure vibration sensor was designed by analyzing the causes of higher-order mode shapes. The impact of this structure on the measurement frequency bandwidth and sensitivity performance of the sensor was investigated through theoretical analysis, simulation verification, sensor fabrication, and experimental testing. 

This article is organized as follows: Section 2 introduces the detailed design of the sensor and the derivation of formulas for the theoretical models. Section 3 describes the verification of the model by the FEM (Finite Element Modeling) method. The fabrication of prototypes is described in Section 4 and experimental validations are presented in Section 5. Finally, Section 6 concludes this work and gives further research directions.

## 2. Materials and Methods

**Figure 1:**
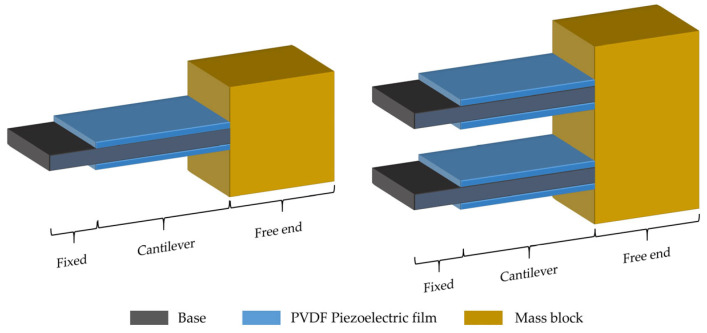
Schematic structure of a single-arm PVDF piezoelectric film cantilever beam and a dual-arm PVDF piezoelectric film cantilever beam.

Traditional single-arm PVDF piezoelectric film cantilever beam structures, as well as dual-arm PVDF piezoelectric film cantilever beam structures, are made up of three parts: the fixed end, the cantilever beam, and the free end [28]. Unlike the single-arm structure, the dual-arm PVDF piezoelectric film cantilever beam consists of two parallel cantilever beams above and below, as shown in Figure 1, wherein the fixed end is rigidly connected by the substrate of the cantilever beam and the fixing component, the cantilever is composed of a piezoelectric material and a substrate, and the free end is composed of a substrate and a mass block.

**Figure 2:**
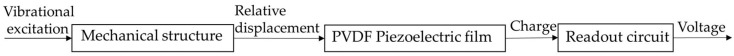
PVDF piezoelectric film cantilever beam vibration sensor design flow chart.

The fixed end is fastened to the vibration exciter plane through a pedestal. When the exciter plane vibrates, the fixed end drives the free end to produce a relative displacement, thereby causing stress of the PVDF piezoelectric film and generation of charges on its surface. The charge is ultimately converted to voltage through the read-out circuit. In accordance with Figure 2, the designed sensor process consists of three parts: the mechanical part, which is a second-order inertial system that determines the relationship between the relative displacements of the fixed end and the free end based on different vibration frequencies; the charge generation part, which involves straining the piezoelectric material due to the relative displacement to generate charge; and the readout circuit part, which converts the charge into a voltage.

### 2.1. Mechanical Structure

**Figure 3:**
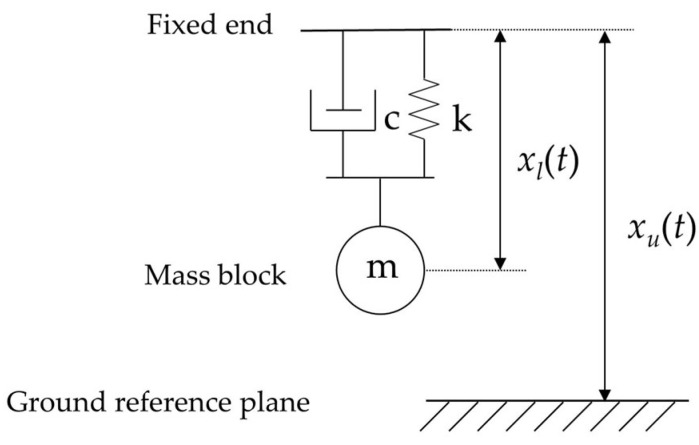
Equivalent mass–spring–damper model.

As shown in Figure 3, the cantilever beam structure is essentially a second-order inertial system composed of a mass block, a spring, and a damper [29]. When the vibration exciter plane generates vibrational excitation *x_u_*(*t*), the free end of the cantilever beam undergoes relative displacement *x_l_*(*t*) with respect to the fixed end due to inertia, where k represents the stiffness coefficient of the cantilever beam structure, c represents the damping coefficient, and m represents the mass of the mass block. According to Newton’s second law and after performing a Laplace transform, the transfer function between the relative displacement *X_l_*(*s*) and the fixed end displacement *X_u_*(*s*) can be obtained as follows:

(1) $$G(s)=\frac{X_{l}\left(s\right)}{X_{u}\left(s\right)}=\frac{s^{2}}{s^{2}+\frac{c}{m}s+\frac{k}{m}}$$

**Figure 4:**
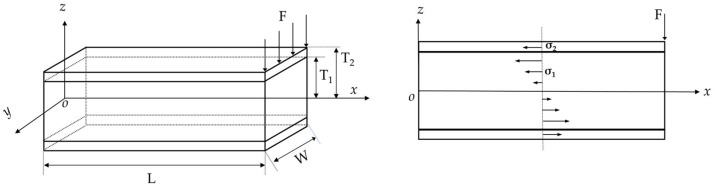
Simplified dimensional model of a single-arm PVDF piezoelectric film cantilever beam and transverse stress distribution diagram.

Figure 4 shows a simplified dimensional model of a single-arm PVDF piezoelectric film cantilever beam. The length of the single-arm PVDF piezoelectric film cantilever beam is denoted as L, the width as w, the base thickness as 2T_1_, and the thickness of the PVDF piezoelectric film as T_1_–T_2_. E_1_ represents the elastic modulus of the base, *σ_1_* represents the stress in the base, E_2_ represents the elastic modulus of the piezoelectric thin film, and *σ_2_* represents the stress in PVDF piezoelectric film. d_31_ is the piezoelectric constant for PVDF piezoelectric film. *x_l_*(*x*) represents the displacement of a point on the *x*-axis of the cantilever beam in the *z*-direction when it is in equilibrium and *x_l_* represents the relative displacement between the fixed end and the center of mass.

Since the resultant force ΣF and resultant moment ΣM of all forces and moments acting on either a rigid or deformable body in equilibrium are both zero (ΣF = 0, ΣM = 0) [30], the following equation is derived, where the left part represents the moment due to internal stresses and the right part represents the moment due to external forces:

(2) $$2\int_{T_{1}}^{T_{2}}\int_{-\frac{W}{2}}^{\frac{W}{2}}\sigma_{2}zdzdy+2\int_{0}^{T_{1}}\int_{-\frac{W}{2}}^{\frac{W}{2}}\sigma_{1}zdzdy=-F(L-x)$$

when a load is applied to a beam, its longitudinal axis is deformed into a curve; the resulting strains *ε* and stresses in the beam are directly related to the curvature *κ* of the bending beam [30]:

(3) $$\sigma_{1}=E_{1}\varepsilon =-E_{1}\kappa z\;\sigma_{2}=E_{2}\varepsilon =-E_{2}\kappa z$$

Substituting Equation (3) into Equation (2) gives the expression:

(4) $$\frac{2W}{3}\left[E_{2}(T_{2}^{3}-T_{1}^{3})+E_{1}T_{1}^{3}\right]\kappa =F(L-x)$$

The formula above is the moment–curvature equation of a piezoelectric film cantilever, where the flexural rigidity is

(5) $$\mathrm{EI}=\frac{2W\left[E_{2}(T_{2}^{3}-T_{1}^{3})+E_{1}T_{1}^{3}\right]}{3}$$

By combining $\kappa =\frac{d^{2}x_{l}(x)}{dx^{2}}$ [30] with Equations (4) and (5), one can obtain:

(6) $$\frac{d^{2}x_{l}(x)}{dx^{2}}\mathrm{EI}=F(L-x)$$

Given the boundary condition at *x*(0) = 0, the solution of Equation (6) is:

(7) $$x_{l}(x)=\frac{F}{\mathrm{EI}}(\frac{L}{2}x^{2}-\frac{x^{3}}{6})$$

when *x* = L, the relationship between the force F and the displacement at the end of the beam *x_l_*(L) is as follows:

(8) $$F=\frac{3\mathrm{EI}}{L^{3}}x_{l}(L)=\frac{2W\left[E_{2}(T_{2}^{3}-T_{1}^{3})+E_{1}T_{1}^{3}\right]}{L^{3}}x_{l}\left(L\right)$$

**Figure 5:**
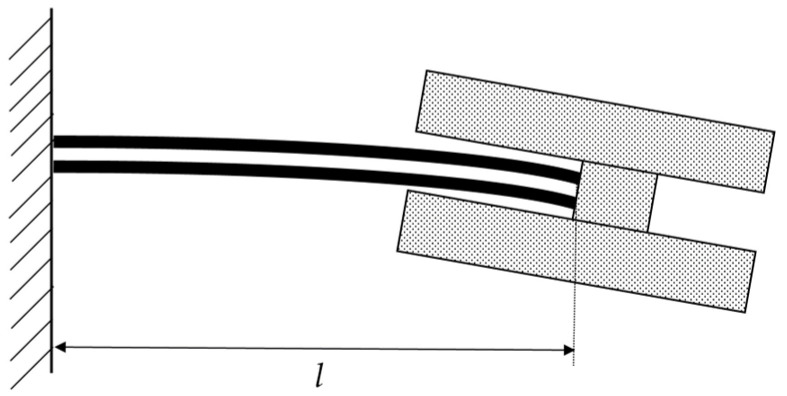
First-mode shape diagram of a single-arm PVDF piezoelectric film cantilever beam.

The location of the mass block of the single-arm piezoelectric cantilever is adjusted to place the center of mass at the cantilever’s end by inserting a shim at the end *x_l_ = x_l_*(L), shown in Figure 5. As a result, the single-arm cantilever structure’s stiffness coefficient k_1_ can be expressed as follows:

(9) $$k_{1}=\frac{F}{x_{l}\left(L\right)}=\frac{2W\left[E_{2}\left(T_{2}^{3}-T_{1}^{3}\right){+E}_{1}T_{1}^{3}\right]}{L^{3}}$$

The first-order natural frequency of a single-arm PVDF piezoelectric cantilever beam structure f_01_ can be derived as:

(10) $$f_{01}=\frac{1}{2\pi }\sqrt{\frac{k_{1}}{m_{1}}}=\frac{1}{2\pi }\sqrt{\frac{2W\left[E_{2}(T_{2}^{3}-T_{1}^{3})+E_{1}T_{1}^{3}\right]}{m_{1}L^{3}}}$$

**Figure 6:**
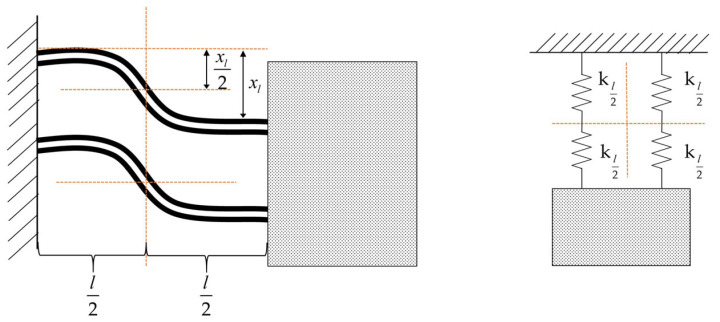
First-mode vibration shape and equivalent spring connection diagram of a double-arm piezoelectric film cantilever beam.

The double-arm PVDF piezoelectric film cantilever beam structure’s first-nature vibration pattern is shown in Figure 6, where each cantilever beam can be equated to two single-arm cantilever beam structures connected in series, the two complete cantilever beams are connected in parallel, and the dual-arm PVDF piezoelectric film cantilever beam coefficient of strength k_2_ can be expressed as:

(11) $$k_{2}=2\times \frac{1}{2}\times \frac{2W\left[E_{2}\left(T_{2}^{3}-T_{1}^{3}\right){+E}_{1}T_{1}^{3}\right]}{{\left(\frac{L}{2}\right)}^{3}}=\frac{16W\left[E_{2}\left(T_{2}^{3}-T_{1}^{3}\right){+E}_{1}T_{1}^{3}\right]}{L^{3}}$$

The first-order natural frequency of a double-arm PVDF piezoelectric cantilever beam structure f_02_ can be derived as:

(12) $$f_{02}=\frac{1}{2\pi }\sqrt{\frac{k_{2}}{m_{2}}}=\frac{1}{2\pi }\sqrt{\frac{16W\left[E_{2}(T_{2}^{3}-T_{1}^{3})+E_{1}T_{1}^{3}\right]}{m_{2}L^{3}}}$$

### 2.2. Piezoelectric Effect

Transverse stress is created by the relative movement of the piezoelectric film cantilever beam’s free and fixed ends. By combining Equations (3), (6), and (8), the stress distribution σ_2_ on the PVDF piezoelectric film with the end load F can be expressed as:

(13) $$\sigma_{2}=E_{2}\varepsilon =E_{2}\kappa z=E_{2}z\frac{F(L-x)}{\mathrm{EI}}=\frac{3E_{2}F\left(L-x\right)z}{2W\left[E_{2}\left(T_{2}^{3}-T_{1}^{3}\right)+E_{1}T_{1}^{3}\right]}$$

According to the piezoelectric effect, the electrical charge density q is equal to the product of the unit stress σ and the piezoelectric constant d_31_, q = d_31_σ [31]. The charge Q generated on the surface of the film is the integral of q over the area; in the structure, it can be expressed as:

(14) $$Q=\frac{W}{T_{1}-T_{2}}\int_{0}^{L}\int_{T_{1}}^{T_{2}}\sigma_{2}d_{31}dzdx=\frac{3E_{2}d_{31}L^{2}\left(T_{1}+T_{2}\right)}{8\left[E_{2}(T_{2}^{3}-T_{1}^{3})+E_{1}T_{1}^{3}\right]}F$$

The parallel connection is utilized to link the top and bottom piezoelectric films as it enhances the total capacitance and equals the voltage distribution of each piezoelectric material, bringing about an increase in output charge [32]. The total charge Q_1_ generated on a single arm piezoelectric cantilever beam utilizing parallel connection versus the relative displacement x*_l_* of the end of the cantilever beam is provided by the expression:

(15) $$Q_{1}=2\times \frac{3E_{2}d_{31}L^{2}\left(T_{1}+T_{2}\right)}{8\left[E_{2}(T_{2}^{3}-T_{1}^{3})+E_{1}T_{1}^{3}\right]}\times \frac{2W\left[E_{2}\left(T_{2}^{3}-T_{1}^{3}\right)+E_{1}T_{1}^{3}\right]}{L^{3}}x_{l}(L)=\frac{3E_{2}d_{31}W(T_{1}+T_{2})}{2L}x_{l}\left(L\right)$$

As shown in Figure 6, for a two-arm piezoelectric film cantilever beam structure, each cantilever beam can still be equated to two single-arm cantilever beam structures in series, both of lengths L/2. Since the two left and right coefficients of stiffness are equal, the displacement generated in the middle symmetry is half of the end displacement, and connecting the equated four single-armed piezoelectric thin films in parallel produces a total charge Q_2_ with a relative displacement x*_l_* of:

(16) $$Q_{2}=4\times 2\times \frac{3E_{2}d_{31}W(T_{1}+T_{2})}{4\times \frac{L}{2}}\times \frac{x_{l}\left(L\right)}{2}=\frac{6E_{2}d_{31}w(T_{1}+T_{2})}{L}x_{l}\left(L\right)$$

### 2.3. Readout Circuit

**Figure 7:**
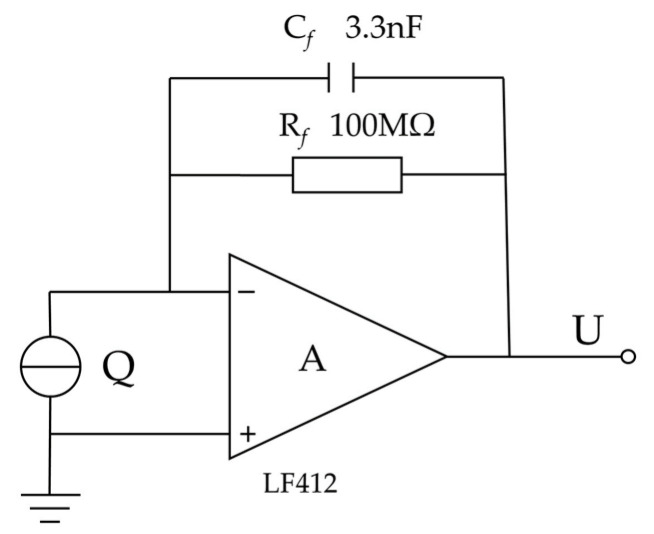
Charge amplifier circuit schematic.

A charge amplifier is created to transform the charge signal into a voltage signal for further processing due to the modest output charge of the piezoelectric thin film cantilever beam, which is generally in the range of tens or hundreds of pC [33]. The schematic diagram of the desired charge amplifier circuit is shown in Figure 7.

A high input impedance of the operational amplifier in the design guarantees efficient conversion of the charge quantity into a voltage signal. The feedback resistor R*_f_* is used to release the charge that has built up in the feedback capacitor C*_f_*, while the feedback capacitor C*_f_* controls the amplifier’s gain. The following is an expression for the relationship between the charge amplifier’s output voltage and the amount of input charge:

(17) $$U=-\frac{Q}{C_{f}}$$

### 2.4. Transfer Function

Given the relative displacement produced by the mechanical component of the piezoelectric thin film cantilever beam structure, the charge generated by the piezoelectric material, and the charge-to-voltage conversion by the charge amplifier, the transfer function between the fixed end displacement *X_u_*(*s*) of the single-arm PVDF piezoelectric film cantilever beam and the output voltage *U*_1_(*s*) of the readout circuit can be written as follows:

(18) $$H_{1}(s)=\frac{U_{1}\left(s\right)}{x_{u}\left(s\right)}=-\frac{s^{2}}{s^{2}+\frac{c_{1}}{m_{1}}s+\frac{k_{1}}{m_{1}}}\times \frac{3E_{2}d_{31}W(T_{1}+T_{2})}{2C_{f}L}=-\frac{S_{1}s^{2}}{s^{2}+\frac{c_{1}}{m_{1}}s+\frac{k_{1}}{m_{1}}}$$

The fixed end displacement *X_u_*(*s*) and readout voltage generation *U*_2_(*s*) transfer function of the dual-arm PVDF piezoelectric film cantilever beam are as follows:

(19) $$H_{2}(s)=\frac{U_{2}\left(s\right)}{x_{u}\left(s\right)}=-\frac{s^{2}}{s^{2}+\frac{c_{2}}{m_{2}}s+\frac{k_{2}}{m_{2}}}\times \frac{6E_{2}d_{31}W(T_{1}+T_{2})}{C_{f}L}=-\frac{S_{2}*s^{2}}{s^{2}+\frac{c_{2}}{m_{2}}s+\frac{k_{2}}{m_{2}}}$$

In Equation (19), S_1_ and S_2_ represent the sensitivity of the single-arm sensor and dual-arm sensor in turn.

(20) $$S_{1}=\frac{3E_{2}d_{31}W(T_{1}+T_{2})}{2C_{f}L},\;S_{2}=\frac{6E_{2}d_{31}W(T_{1}+T_{2})}{C_{f}L}$$

### 2.5. Structural Parameter Design

Manganese steel was chosen as the base due to its high resilience performance, which ensures that there will be no fatigue or damage over time; at the same time, iron also offers advantages such as a high density, a low cost, and ease of processing. Therefore, metal iron was selected as the mass block. Table 1 displays the material parameters.

PVDF piezoelectric films have the following flexibility and coupling matrices:

$$\begin{array}{c} s=\left[\begin{array}{cccccc} 3.78 & -1.48 & -1.72 & 0 & 0 & 0\\ 1.9 & 3.78 & 0.9 & 0 & 0 & 0\\ 0.9 & 0.9 & 10.92 & 0 & 0 & 0\\ 0 & 0 & 0 & 14.28 & 0 & 0\\ 0 & 0 & 0 & 0 & 11.1 & 0\\ 0 & 0 & 0 & 0 & 0 & 11.1 \end{array}\right]*10^{-10}(\frac{1}{\mathrm{Pa}})\\ d=\left[\begin{array}{cccccc} 0 & 0 & 0 & 0 & 0 & 0\\ 0 & 0 & 0 & 0 & 0 & 0\\ 3.2 & 0.47 & -2.6 & 0 & 0 & 0 \end{array}\right]*10^{-11}\left(C/N\right) \end{array}$$

In this study, we built single-arm and dual-arm piezoelectric cantilever beams with a first-natural frequency of 12 Hz to assess the impact of the second-mode shape on the measured bandwidth between the two constructions. The dimensions of the beam were consistent during the cantilever beam testing, and a mass block of size 15 × 15 mm was employed. The mass of the mass block was altered by altering its height, ensuring that the initial natural frequencies of the two structures remained approximately comparable. Table 2 shows the basic parameters of both structures.

The stiffness coefficients for the two constructions may be calculated using Equations (9) and (11) based on the given material qualities and dimensions. The first-order nature frequency of the single-arm structure is 11.7 Hz, while the first-order nature frequency of the dual-arm structure is 11.8 Hz, according to the end block mass. Furthermore, the charge generated by a 1 mm relative displacement may be estimated using Equations (15) and (16). By connecting this formed charge to the charge amplifier, the corresponding voltage produced under a 1 mm displacement may be acquired. The results are shown in Table 3.

## 3. Simulation Verification

In this section, validation of the designed single-arm and dual-arm structures is carried out through modal analysis using the finite element simulation software COMSOL Multiphysics^®^, Version [5.6]. Separate modal analysis simulations were carried out, and the voltage generated by two different piezoelectric materials under the same relative displacement was compared. In addition, using the same input load, a frequency domain analysis was performed to calculate the charge created by the piezoelectric material.

### 3.1. Modal Simulation

**Figure 8:**
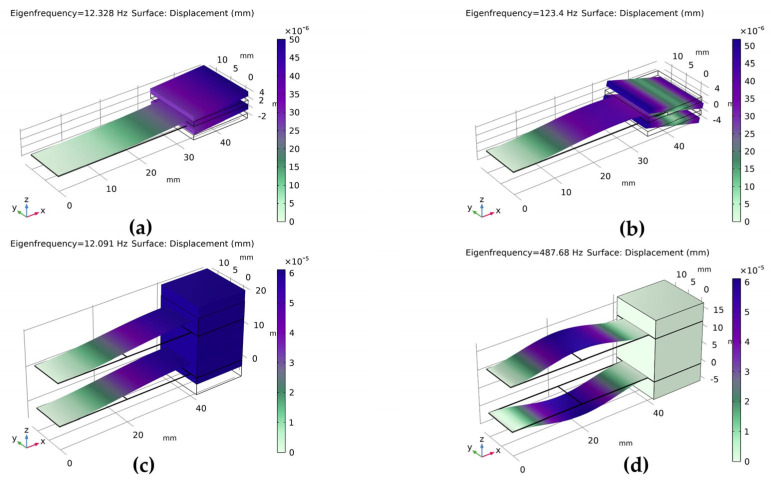
Modal vibration mode contour plots of PVDF piezoelectric film cantilever beams: (**a**) first-mode shape of the single-arm structure; (**b**) second-mode shape of the single-arm structure; (**c**) first-mode shape of the dual-arm structure; (**d**) second-mode shape of the dual-arm structure.

During the analysis of characteristic frequencies, the leftmost fixed end of the cantilever beam was constrained. The modal shapes of the first and second modes for both single-arm and dual-arm cantilever beam structures are obtained, as shown in Figure 8. The finite element simulations indicated that the dual-arm construction has a much higher second-order natural frequency than the single-arm structure at the same first-order natural frequency.

### 3.2. Steady-State Charge Simulation Comparison

**Figure 9:**
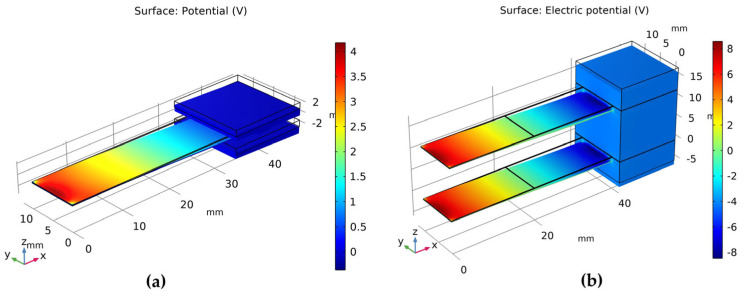
Potential distribution contour plots of PVDF piezoelectric film cantilever beams: (**a**) potential distribution of single-arm structure; (**b**) potential distribution of dual-arm structure.

In the steady-state analysis, the fixed end was constrained and the contact surface between the piezoelectric material and the base was grounded. The charge created during the vibration process was then simulated by applying an upward displacement of 1 mm at the centroid of the free end with a relative displacement of 1 mm. The simulated potential contour plot is shown in Figure 9.

From the derived results, the charge generation in the piezoelectric material was determined by calculating the average voltage on its surface. Since the charge is equal to the product of capacitance and surface voltage, the amount of charge generated in the piezoelectric material can be obtained. The results are shown in Table 4.

## 3.3. Frequency Domain Analysis

**Figure 10:**
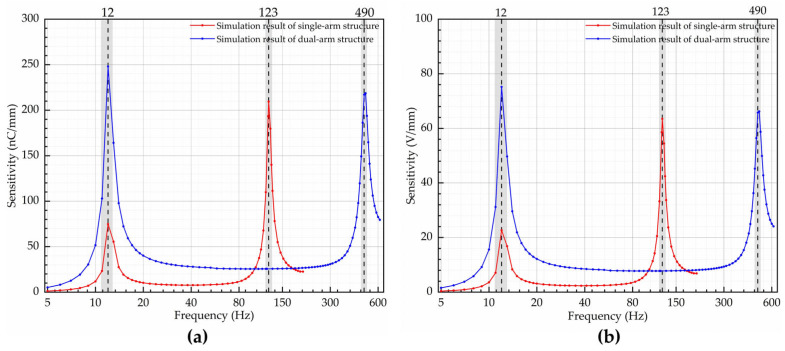
Simulation results of charge and voltage generation for two structures under different frequency sinusoidal excitations: (**a**) charge generated on the surface of the piezoelectric material without being connected to a charge amplifier; (**b**) voltage generated when connected to a charge amplifier.

When performing the frequency domain analysis, the fixed end of the piezoelectric cantilever beam structure was secured. A damping factor of 0.08 was applied to the single-arm structure, and a damping factor of 0.1 was applied to the dual-arm structure. The damping factors were determined through the physical fabrication and testing of the two structures, and the coefficients were obtained through fitting. To simulate the vibration signals at the fixed end of the cantilever structure, the two structures were injected with volume load signals at varying frequencies. Finally, the relationship between the charge generated by the piezoelectric material under the influence of vibration signals with the same amplitude but different frequencies for the two different structures was obtained. This charge was then fed into the designed charge amplifier to generate voltage, as depicted in Figure 10.

## 4. Fabrication

**Figure 11:**
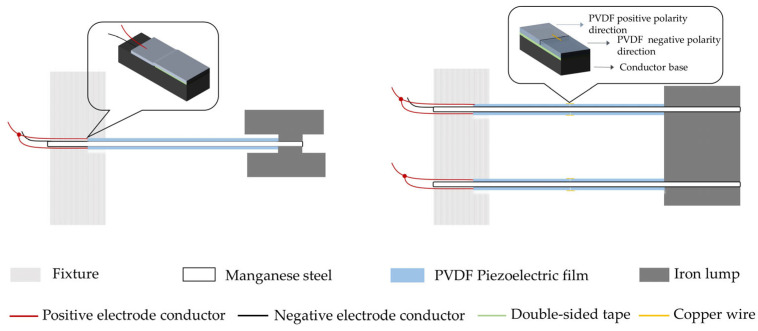
The schematic diagram of connection process of the PVDF piezoelectric film cantilever beam.

**Figure 12:**
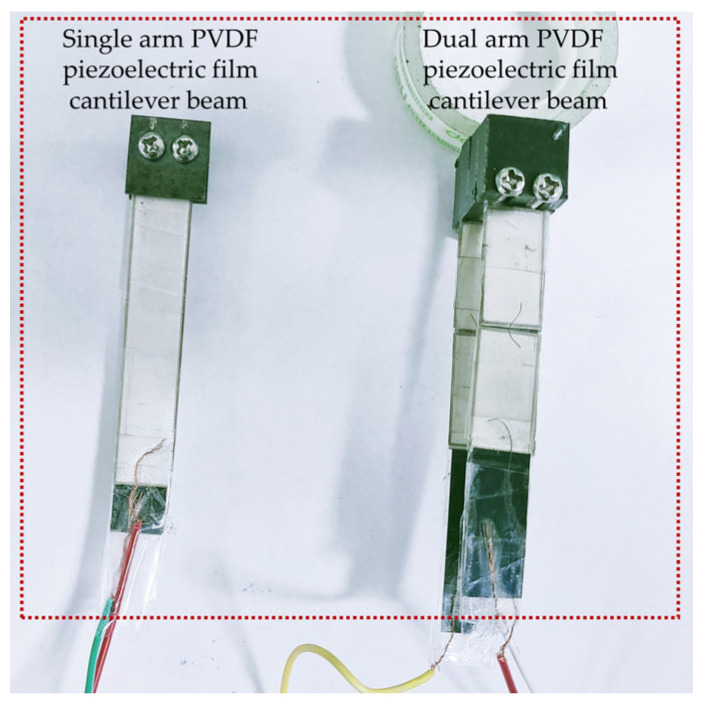
PVDF piezoelectric film cantilever beam fabrication result.

The following describes the sample preparation procedure: The PVDF piezoelectric film was first attached to the base using a flexible dual-sided adhesive with a thickness of around 0.015 mm. To provide a rigid connection between the iron block and the hung beam, free end bolts were used. The fixed end was clamped with a fixture and secured with bolts. In order to ensure that the polarities of the PVDF piezoelectric films connected in parallel were the same, it was necessary to attach piezoelectric films with a positive polarity to both the upper and lower sides of the same cantilever beam on the base. The transverse stresses created by the left and right cantilevers were in opposing directions; thus, it was important to attach a piezoelectric material in the opposite polarity direction when creating the dual-arm PVDF piezoelectric film cantilever beam. In order to complete the parallel connection of the piezoelectric film’s positive electrode, metal wire was used to contact the film’s surface. Similarly, a metal base and a metal mass block were used as conductors to complete the parallel connection of the piezoelectric film’s negative electrode. The final connection result and the fabrication outcome are shown in Figure 11 and Figure 12, respectively.

## 5. Experiment

### 5.1. Test Platform

**Figure 13:**
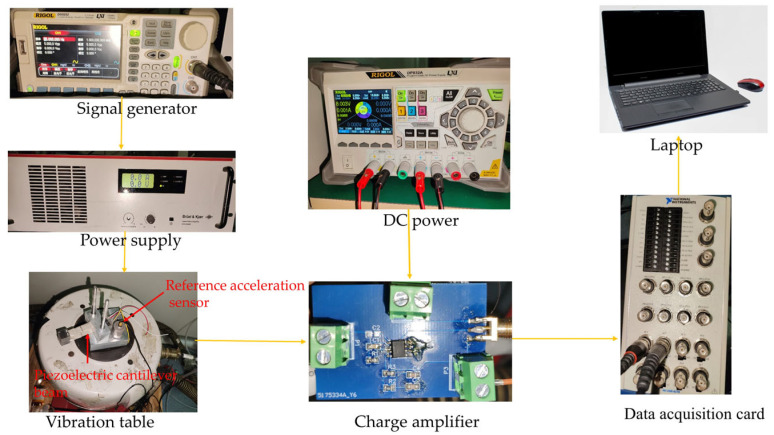
Experimental test procedure flowchart.

**Figure 14:**
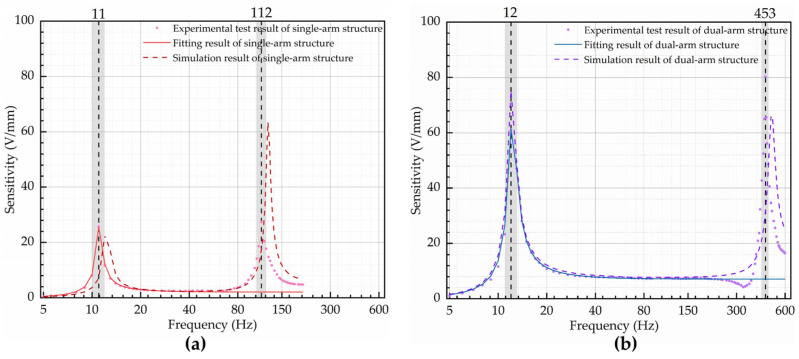
Experimental testing, fitting, and simulation results of the frequency response for two structural configurations: (**a**) single-arm structure; (**b**) dual-arm structure.

The main components included in the test system are a Rigol DP831A DC power supply, a Rigol DG5252 signal generator, a Bruel & Kjaer LDS LPA600 power amplifier, a Bruel & Kjaer LDS VIBRATOR V450 vibration table, a Bruel & Kjaer 4370 V reference accelerometer, and an NI USB-6216 data acquisition card. Figure 13 shows the test procedure flowchart, in which the signal generator is used to output a sinusoidal excitation signal. The power amplifier amplifies and transmits the power to the vibration table, and the vibration table generates vertical vibration at a given frequency. The PVDF piezoelectric film cantilever beam vibration sensor and the reference accelerometer are placed on the same vibration plane. The charge signal generated by the PVDF piezoelectric film cantilever beam vibration sensor is converted to a voltage signal through a charge amplifier, which is then collected by the data acquisition card and sent to the notebook computer for processing to obtain the output voltages of the two sensors. In this test experiment, the sinusoidal sweeping excitation frequency of the vibration table was in the range of 5 Hz to 598 Hz, and the measurement frequency intervals are shown in Figure 14.

### 5.2. Test Results

Experiments on both structures were performed to calibrate the sensitivity, damping coefficient, and natural frequency, which are the parameters of a second-order inertial system transfer function. In this experimental test, for the single-arm structure, the test range was 5 Hz to 202 Hz and the fitted range was 5 Hz to 68 Hz; for the dual-arm structure, the test range was 5 Hz to 598 Hz and the fitted range was 5 Hz to 270 Hz. The test results and fitted curves are shown in Figure 14.

A comparison between the experimental test fitting results and the theoretical calculations and simulations for the two structures in this experiment is presented in Table 5.

In this study, a comparison was made between the traditional single-arm cantilever piezoelectric film structure and the designed dual-arm cantilever piezoelectric thin film structure in terms of the first-order natural frequency, second-order natural frequency, measurement bandwidth, and sensitivity. The specific comparison results are shown in Table 6.

## 6. Conclusions

In this study, we performed integrated mathematical modeling of cantilever-beam-type piezoelectric film sensing and designed a dual-arm piezoelectric thin film sensor. Finite element simulations were performed to validate the design, followed by fabrication and experimental testing. There was ample agreement between the measured, simulated, and computed results. The results of the experiment demonstrate that the proposed dual-arm structure vibration sensor largely retains its first-order natural frequency (around 12 Hz) in contrast to the conventional single-arm structure vibration sensor. In addition, the sensitivity rose by a factor of 3.48, and the second-order natural frequency increased from 112 Hz to 453 Hz. For the dual-beam construction, the ratio of the second-order natural frequency to the first-order natural frequency is around 36.8, beyond the conventional limit of roughly 10 to 20.

The widening of the measurement bandwidth may be due to the fact that the mass blocks of the dual-arm structure can move vertically up and down without premature bending deformations due to their excessive weight. Thus, while the first natural vibration frequency is consistent, the second natural frequency is significantly increased. Furthermore, the strain distribution of the piezoelectric film at both ends of the dual-beam structure has been modified, allowing for greater charge transfer and higher sensitivity.

In the conducted experimental tests, the dual-arm structure exhibited a significantly lower sensitivity before the second-order frequency compared to the predicted sensitivity. This could be attributed to the superposition of the two vibration modes, causing charge cancellation and resulting in reduced sensitivity.

Further research we are considering carrying out includes (1) investigating the detection bandwidth versus the natural frequency by testing more dual-arm prototypes with varied natural frequencies; (2) fabricating highly consistent prototypes and conducting field experiments; and (3) investigating gravity effects on sensor performance (e.g., linearity).

## Figures and Tables

**Table 1:** Material parameters.

	Young’s Modulus (Pa)	Density (kg/m^3^)	Poisson Ratio	Relative Dielectric Constant
Manganese steel	198 × 10^9^	7800	0.35	/
PVDF	2.8 × 10^9^	1780	/	13.5
Iron	198 × 10^9^	7800	0.35	/

**Table 2:** Basic parameters of both structures.

	Single-Arm Structure	Dual-Arm Structure
Cantilever material	manganese steel	manganese steel
Cantilever length (mm)	40	40
Cantilever width (mm)	10	10
Base thickness (mm)	0.15	0.15
PVDF thickness (mm)	0.028	0.028
Arm distance (mm)	/	12
Block material	iron	iron
Block size (mm^3^)	15 × 15 × 2.6	15 × 15 × 20.8
Block mass (g)	4.88	38.61

**Table 3:** Theoretical calculation results of two cantilever beam structures.

	First-Order Natural Frequency (Hz)	Generated Charge (C) by a 1 mm Relative Displacement	Voltage after Connecting Charge Amplifier (V)
Single-arm structure	11.7	5.78 × 10^−9^	1.9
Dual-arm structure	11.8	23.07 × 10^−9^	7

**Table 4:** The average surface voltage and total charge generated by simulation for a 1 mm displacement of the PVDF piezoelectric film.

	Voltage (V)	Charge (C)
Single-arm structure	1.84	6.28 × 10^−9^
Dual-arm structure	3.68	25.13 × 10^−9^

**Table 5:** Results of the comparison between the two structures.

	Structure	Theory	Simulation	Experiment
First-order|Second-order natural frequency (Hz)	Single arm	11.7|/	12.3|123.4	11|112
	Dual arm	11.8|/	12|487.8	12.3|453
Sensitivity (V/mm)	Single arm	1.75	1.90	2.05
	Dual arm	7	7.62	7.12

**Table 6:** Comparison of experimental test results for the two structures.

	Measurement Band with ±20% Tolerance (Hz)	Ratio of Second-Order Natural Frequency to First-Order Natural Frequency	Sensitivity (V/mm)	Ratio of Dual-Arm Sensitivity to Single-Arm Sensitivity
Single arm	27–65	10.2	2.05	——
Dual arm	29~280	36.8	7.12	3.48

## Data Availability

The data presented in this study are available on request from the corresponding author.
